# Cultural adaptation and validation of the Norwegian version of the swallowing quality of life questionnaire (SWAL-QOL)

**DOI:** 10.1186/s12955-019-1248-0

**Published:** 2019-12-05

**Authors:** Maribeth Caya Rivelsrud, Melanie Kirmess, Lena Hartelius

**Affiliations:** 1 University of Gothenburg, Gothenburg, Sweden; 2 Department of Research, Sunnaas Rehabilitation Hospital, Bjørnemyrveien 11, 1453 Bjørnemyr, Norway; 3 Department of Special Needs Education, University of Oslo, Oslo, Norway; 4 Department of Health and Rehabilitation, Institute of Neuroscience and Physiology, University of Gothenburg, Gothenburg, Sweden

**Keywords:** Deglutition disorders, Oropharyngeal dysphagia, Health-related quality of life, SWAL-QOL, Translation, Psychometric, Assessment, Treatment

## Abstract

**Introduction:**

Oropharyngeal dysphagia (OD) is a disorder that can have devastating and long lasting effects on a person’s medical, mental and psychosocial well-being, thus negatively impacting quality of life. There is currently no validated dysphagia-specific quality of life instrument in Norway. This project aims to evaluate the psychometric properties of the culturally adapted Norwegian version of SWAL-QOL (Nor-SWAL-QOL).

**Methods:**

The original SWAL-QOL was translated into Norwegian according the international translation guidelines. A group of 102 persons with OD and a group of 123 healthy controls were recruited to assess the validity and reliability of the Nor-SWAL-QOL. Correlation analysis of the Nor-SWAL-QOL and the Short Form 36 (SF-36) and correlation analysis of OD group and control group Nor-SWAL-QOL subscale scores were computed to determine convergent, discriminant, and known-groups validity which help comprise construct validity. Internal consistency, test-retest reliability and intraclass correlation coefficient (ICC) were computed for reliability.

**Results:**

Convergent and discriminant validity was demonstrated between Nor-SWAL-QOL subscales and SF-36 domains, and distinguished between persons with and those without oropharyngeal dysphagia on all subscales and on the symptom frequency battery (*p < 0.001).* Additionally, the Nor-SWAL-QOL differentiated between symptom severity levels within the OD group; those requiring food and liquid modifications and those who are tube fed and not tube fed. Nor-SWAL-QOL showed good reliability with adequate internal consistency (Cronbach’s α ≥0.70), test-retest reliability (Spearman’s rho values 0.68–0.90) and ICC values (0.67–0.89) for all subscales and for the symptom frequency battery.

**Conclusion:**

Access to valid and reliable dysphagia-specific QoL outcome measures for health care practitioners, dysphagia clinicians and researchers is necessary for comprehensive assessment and treatment outcome measures. The Nor-SWAL-QOL exhibits sufficient psychometric properties for implementation in the Norwegian population.

## Background

Oropharyngeal dysphagia (OD) is a disorder affecting over 40 million people in Europe [1] and is particularly common in the elderly, persons with neurological disorders or disease and those treated for head and neck cancer. Literature reporting the prevalence of OD vary between 5 and 72% for non-institutionalized elderly [2], 8–80% in acute stroke [1] and 55–75% for head and neck cancer [3]. These variations are often due to differences in etiology, definitions of dysphagia, study design, timing and type of assessment (screening, clinical or instrumental).

Common symptoms of OD are coughing and choking, complaints of food sticking in the throat, drooling, prolonged mealtimes and unexplained weight loss [4]. OD is known to be a significant predictive factor for malnutrition, dehydration and pneumonia. Medical complications resulting from OD are associated with morbidity, prolonged hospital stay and increased health care costs [5–7]. The management of swallowing symptoms often includes modifications of food and liquid consistencies, and in severe OD enteral feeding may be necessary, affecting quality of life (QoL) [8, 9]. OD is known to have lasting psychosocial impacts on QoL associated with increased fear, embarrassment, anxiety, depression and social isolation [10–12].

Despite knowledge of OD having a devastating impact on an individual’s health and well-being, OD is unfortunately an under-diagnosed, under-reported and a poorly managed disorder [1, 10, 13]*.* Many dysphagia sufferers and healthcare professionals are not even aware that dysphagia is treatable [10].

The use of objective clinical and instrumental assessments, such as the Mann Assessment of Swallowing Ability (MASA), Functional Oral Intake Scale (FOIS) and the videofluoroscopic swallow study (VFSS), to determine the physiological severity of OD and treatment alternatives is well reported in the literature [14–16]. However, healthcare personnel and dysphagia clinicians’ perspectives about the physiological consequences of a disorder may deviate considerably from the patients’ perspectives on living with a disorder [17]. This observation has resulted in increased development and use of patient-reported outcome measures (PROM) and disease-specific health-related quality of life (HRQoL) instruments.

There is currently no validated HRQoL instrument available in Norwegian for the documentation of patient perspectives on living with oropharyngeal dysphagia. The primary purpose of this project was to complete a cultural adaptation, validation and reliability study of the SWAL-QOL for a Norwegian population and answer the following research questions based on the original SWAL-QOL; can a Norwegian version of SWAL-QOL 1) demonstrate reliability and 2) demonstrate construct validity; (i.e. show convergent and discriminant validity, distinguish between persons with and those without oropharyngeal dysphagia, be sensitive to clinically known features of oropharyngeal dysphagia, and be able to discriminate between symptom severity levels). It is hypothesized that the OD group will score lower than control group on all domain and symptom frequency battery scores. It is also hypothesized that within the OD group, persons having modified consistencies of food and liquid, and tube feeding will score lower than their peers on normal consistency and not tube fed.

## Methods

### Cultural adaptation of the Nor-SWAL-QOL

The SWAL-QOL questionnaire was one of the first patient-based tools developed for use in both research and clinical practice to illuminate how QoL is affected by dysphagia [18–20]. The author’s definition of QoL used in instrument development was adapted from Gotay et al. [21] and includes: a) the ability to fulfill usual and desired physical, role, and social activities, b) the psychological effectiveness with which one performs usual and desired activities, c) dysphagia symptom status and d) satisfaction with health care services related to dysphagia treatment. This final point was used in the development of SWAL-CARE which is not included in this study [18].

SWAL-QOL is a 44 item tool that takes on average 15 min to complete and assesses 10 quality of life concepts; eight of which are dysphagia-related QoL (food selection, burden, mental health, social functioning, fear, eating duration, eating desire, communication) and two pertaining to general QoL (sleep and fatigue). The questions are intended to reflect the swallowing problem experience within the preceding month. The SWAL-QOL also includes a symptom frequency battery of 14 questions, three questions regarding type of nutritional intake and one question on general health. The questions are scored on a 5-point Likert scale which can be transformed to achieve scores ranging from 0 (least favorable state) to 100 (most favorable state). The questionnaire concludes with general information questions including age, gender, education, marital status and need for assistance and amount of time used to complete the questionnaire. SWAL-QOL has been translated and validated in several languages including Dutch, Swedish, Italian, French, German and Chinese [22–28].

For this study, the original SWAL-QOL was translated into Norwegian based on guidelines described by Beaton and colleagues [29]. Two individuals, proficient in both English and Norwegian and knowledgeable in dysphagia, completed independent translations of the original SWAL-QOL into Norwegian (stage 1: forward translation). These two translators discussed discrepancies in their translations working from the original and adjustments were made for general questions in order to reflect cultural differences for classification of race/ethnicity, education and civil status. The original author was contacted for clarifications on possible changes in wording and the two translators agreed on a first draft of the Norwegian version. This draft was sent to an interdisciplinary panel consisting of a doctor, nurse, neuropsychologist, sociologist and occupational therapist and two lay persons. These comments were compiled and the two translators came to a consensus on the final version to be sent for back-translation (stage 2: synthesis). The final Norwegian version was back-translated to English by an authorized translator without access to the original questionnaire (stage 3: back-translation). An expert committee review was comprised of the original translators, authorized back-translator, two interdisciplinary panel members with experience in cultural adaptation and translation and two user representatives familiar with dysphagia. The expert committee reviewed the original version, back translation and Norwegian version, evaluating the semantic, idiomatic, experiential and conceptual equivalence (stage 4: expert committee review). Finally, this Norwegian version of the SWAL-QOL (Nor-SWAL-QOL) was piloted with seven persons with subjective oropharyngeal dysphagia and seven without subjective oropharyngeal dysphagia. Each participant was interviewed individually and asked if they felt the translation was difficult to answer, confusing, difficult to understand or upsetting/offensive (stage 5: pre-testing). A report with the description of the cultural adaptation and translation process was submitted and approved by the original developer of the SWAL-QOL.

### Study participants

Adults assessed for OD at Sunnaas Rehabilitation Hospital between January 2014–February 2018 were considered eligible for this study (*n = 305*). Sunnaas is the largest Rehabilitation hospital in Norway, with both in and outpatient services for all of Norway. All patients had been clinically assessed for OD with the Mann Assessment of Swallowing Ability (MASA) and undergone a videofluoroscopic swallow study (VFSS). A pre-approved project information and consent letter from the Regional Ethics Committee was either sent in the mail or given directly to the 305 eligible participants. Accompanying the information and consent letter were two questionnaires (Short Form-36 survey of health, SF-36 and Nor-SWAL-QOL). In addition, a personalized cover letter was included with detailed information about how to fill out and return the forms with the intention to participate and included an option to return only the cover letter to signify non-participation. The purpose of this was to document eventual reasons for non-participation (no swallowing difficulties or other). Missing respondents received a reminder via post and/or telephone after 2–3 weeks.

A priori sample size of 120–150 was determined by “rule of thumb” recommending 12–15 observations per domain [30].

Of the 305 eligible participants, 231 persons responded to the survey, of which 103 replied that they did not wish to participate, resulting in 128 participants. Inclusion criteria were: 1) ≥18 years of age and 2) stable dysphagia ≥2 months. Exclusion criteria included: 1) inability to provide informed consent, 2) inability to understand written or spoken Norwegian, 3) denial of dysphagia by the patient, and 4) evidence of pure esophageal dysphagia. Seven persons did not meet these inclusion criteria, thus 121 patients were originally included in the study. Nineteen of these had > 10% missing answers on the Nor-SWAL-QOL and were excluded from further statistical analysis, resulting in 102 OD cases.

A control group of 123 individuals without neurological illness/injury or subjective oropharyngeal or esophageal dysphagia were recruited via project announcements on social media, snowballing and project presentations at patient/caregiver organization meetings. All participants provided informed consent and completed the Nor-SWAL-QOL and SF-36.

Every other participant was asked to complete the Nor-SWAL-QOL a second time, approximately 2 weeks following the initial administration, in order to assess test-retest stability. Thirty-four participants returned the questionnaires within 2–3 weeks.

### Short Form-36

The Short Form-36 (SF-36) is one of the most internationally used questionnaires for quality of life in the world. SF-36 is a patient-reported survey for general health status containing 36 questions covering eight health concepts: physical functioning, role limitations due to physical health problems, role limitations due to emotional problems, social functioning, emotional well-being, energy/fatigue, pain and general health perceptions. It utilizes a standardized scoring system which can be transformed into a 0–100 scale (0 = worst possible, 100 = best possible HRQoL). The SF-36 was used to test validity in the original SWAL-QOL development and has been used frequently in other cultural adaptation and validation studies of the SWAL-QOL. The Norwegian version has well documented reliability and validity and has been used in Norway since 1998 [31].

### Functional Oral Intake Scale

The Function Oral Intake Scale (FOIS) is a 7-point ordinal scale developed for health care professionals’ use in documenting current functional oral intake of patients with dysphagia and change of oral intake over time [14]. The FOIS may be completed through patient observation, review of medical journal, dietary journals or information provided by the patient or caregiver. The seven levels of functioning are subdivided into 2 sections where levels 1–3 include varying degrees of non-oral feeding and levels 4–7 include varying degrees of oral feeding without non-oral supplementation. The FOIS is reported to be a valid and reliable instrument for use with acute stroke patients and is utilized with other patient populations in the international literature [14, 32, 33]. In this study, the FOIS score was established for each OD participant following a telephone interview by the first author. This interview aided in gathering more specified information about status of oral intake (oral and/or use of feeding tube), need for compensatory adjustments, inquire about the reason for eventual missing items and to express appreciation for their participation in the study. The FOIS levels for OD participants are provided in the Additional file 1.

### Statistical analysis

Statistical analysis was performed using IBM SPSS Statistics for Windows, version 23 (IBM Corp., Armonk, N.Y., USA). Non-parametric tests were used where appropriate as the data was determined to be not normally distributed by Shapiro-Wilk and Kolmogorov-Smirnov tests of normality. A weighted adjustment was completed for variables of age and gender between the OD and control groups in this study sample. Correlation analysis of results from the OD group on the Nor-SWAL-QOL and SF-36 using Spearman’s rho (r_s_) was calculated for determining convergent and discriminant validity. Bonferroni-correction was used for these multiple correlations. A principal component analysis (PCA) was performed in order to identify underlying components and explain the maximal amount of total variance in the data using the fewest number of explanatory constructs. The sample size was determined to be sufficient for PCA with a KMO value of 0.805 (> 0.6) and a statistically significant Bartlett’s Test of Sphericity of *p* < 0.001. Oblique rotation (Oblimin) was performed in addition to orthogonal rotation (Varimax) as the components are not assumed to be independent [34, 35].

Correlations of OD group and control group Nor-SWAL-QOL scale scores were computed for known-groups validity using Mann-Whitney U. The effect size was determined to demonstrate clinical relevance for group comparisons. Cronbach’s α was computed for reliability for internal consistency of Nor-SWAL-QOL subscales and symptom frequency battery, and Spearman’s rho, two-way mixed model for absolute agreement, single rater, was computed for test-rest reliability and intraclass correlation coefficient (ICC). All tests were two-tailed and conducted at α = 0.05 level.

## Results

### Descriptive data

#### Participant characteristics and feasibility of the Nor-SWAL-QOL

Total recruitment response rate for the study was 75.7%, of which 55% participated. The study group included in the analysis was comprised of 58% men (*n* = 59/102) with ages ranging from 24 to 87 years with a mean of 60.6. Etiology of the dysphagia participants was 58% neurological disorders, 15% head and neck cancer, 18% other diagnosis and 10% unknown. Forty-one percent (*n* = 41/100) of participants with OD required some form of help filling out the forms. Seventy-four percent of patients (*n* = 73/99), including those requiring help or not, reported using between 15 and 30 min to complete the Nor-SWAL-QOL.

The time period from OD onset and Nor-SWAL-QOL ranged from 2 months to 44 years. The majority of the study group (90%) had lived with OD for more than 1 year and over half of the study group (62.%) ate a modified diet or were tube fed. The FOIS revealed that only 27/102 (26%) of the study group could eat and drink without restrictions or avoiding specific foods because of their swallowing problems. Tables with the descriptive characteristics of the OD and control groups, and FOIS scale distribution for the study group are available in the Additional file 1.

### Score distribution

Score distributions for the Nor-SWAL-QOL in the OD group covered the full range (0–100) with the exception of the symptom frequency battery (range 14–100) see Table 1. Mean scores ranged from 40.0 (eating duration) to 65.5 (eating desire). Floor effects of > 15% occurred for one scale; eating duration (16.7%), while ceiling effects were observed for 4 scales; food selection, eating desire, sleep and communication (15.7–23.5%).

**Table 1:** Score distributions for Nor-SWAL-QOL for oropharyngeal dysphagia group *(n = 102)*

Nor-SWAL-QOL	Items	Range	Mean	Median	SD	% Floor effects	% Ceiling effects
Burden^a^	2	0–100	44.6	50.0	28.3	10.8	3.9
Food selection	2	0–100	57.3	63.0	31.7	9.8	16.7
Eating duration	2	0–100	40.0	38.0	29.7	16.7	5.9
Eating desire	3	0–100	65.5	75.0	28.9	4.9	15.7
Fear of eating	4	0–100	62.3	63.0	27.6	2.9	11.8
Sleep	2	0–100	60.0	63.0	31.1	8.8	19.6
Fatigue^a^	3	0–100	52.9	58.0	28.2	7.8	5.9
Communication^a^	2	0–100	61.9	75.0	33.0	8.8	23.5
Mental Health	5	0–100	46.3	40.0	28.2	6.9	3.9
Social functioning	5	0–100	54.4	55.0	31.4	6.9	13.7
Symptom frequency battery	14	14–100	55.6	59.0	19.4	2.9	1.0

^a^Number of participants included for these subscales *n = 101*

### Reliability

In order to address the first research question of whether the Nor-SWAL-QOL is reliable, Cronbach’s α was computed for internal consistency of the Nor-SWAL-QOL subscales, and Spearman’s rho, two-way mixed model for absolute agreement, single rater, was computed for test-rest reliability and intraclass correlations coefficients (ICC). ICC values less than 0.5 were considered of poor reliability, from 0.5 and 0.75 moderate reliability, from 0.75 and 0.9 good reliability, and greater than 0.90 excellent reliability [36]. Reliability results are displayed in Table 2. All 10 subscales and the symptom frequency battery of the Nor-SWAL-QOL achieved acceptable Cronbach’s α of > 0.70, all but two subscales (eating duration, eating desire) achieved the recommended Cronbach’s α for group level research of ≥0.80 and communication met the recommended Cronbach’s α > 0.95 for individual-patient assessment [37, 38]. A subset of 34 OD participants with test-retest time interval of 2–3 weeks revealed moderate to strong reliability (0.68–0.90. ICC values ranged from moderate (0.67; 95% CI 0.43–0.82) to good (0.89; 95% CI 0.79–0.95) reliability.

**Table 2:** Reliability estimates for Norwegian version of the Swallowing Quality of Life (Nor-SWAL-QOL)

Nor-SWAL-QOL	Internal consistency^a^ (Cronbach’s α)	*n=*	Test-retest^b^ (Spearman’s *r*_*s*_)	*n=*	Intraclass correlation^c^
Burden	0.85	101	0.68*	34	0.67
Food selection	0.85	102	0.83*	34	0.81
Eating duration	0.73	102	0.74*	34	0.74
Eating desire	0.73	100	0.73*	33	0.75
Fear	0.80	101	0.66*	33	0.67
Sleep	0.82	101	0.82*	34	0.82
Fatigue	0.89	98	0.84*	34	0.85
Communication	0.95	99	0.85*	33	0.88
Mental health	0.91	101	0.77*	34	0.79
Social functioning	0.91	98	0.85*	34	0.84
Symptom frequency battery	0.87	89	0.90*	33	0.89

^a^Internal consistency

^b^Test-retest average time interval 18.5 days; *Correlation is significant at *p* < 0.01

^c^Intraclass correlation coefficient (ICC)

### Validity

#### Construct validity

Validation of a measurement involves determining to which degree an instrument is able to measure what it claims to measure. Assessing the construct validity of an instrument, the degree to which the content is an adequate representation of the construct to be measured is a necessary step in determining validity.

Convergent validity is the degree to which two similar constructs correlate with each other. Conversely, discriminant validity is the degree to which two dissimilar constructs do not correlate with one another. Correlations were considered strong with Spearman’s rho values < 0.7, moderate with values between 0.3–0.7 and weak with values > 0.3 [38, 39].

To address the second question of this study, correlation analysis of results from the OD group on the Nor-SWAL-QOL and SF-36 using Spearman’s rho (r_s_) was calculated for determining convergent and discriminant validity. Results are displayed in Table 3. The Nor-SWAL-QOL subscales burden, food selection, sleep, fatigue, mental health and social functioning showed significant correlations (r_s_ = 0.36–0.71) with several of the SF-36 domains.

**Table 3:** Construct validity of Nor-SWAL-QOL and Short Form 36 in oropharyngeal dysphagia group *(n = 102)*

Nor-SWAL-QOL	SF-36							
	Physical functioning	Role physical	Bodily pain	General health	Vitality	Social functioning	Role emotional	Mental health
Burden	0.19	0.36*	0.23	0.32	0.33	0.38*	0.20	0.30
Food selection	0.14	0.38*	0.31	0.41*	0.38*	0.39*	0.14	0.31
Eating duration	0.30	0.23	0.19	0.28	0.20	0.26	0.29	0.22
Eating desire	0.23	0.17	0.15	0.28	0.28	0.23	0.16	0.36*
Fear of eating	0.22	0.20	0.28	0.30	0.21	0.15	0.14	0.29
Sleep	0.13	0.31	0.42*	0.52**	0.43*	0.42**	0.28	0.36*
Fatigue	0.23	0.42*	0.51**	0.65**	0.71**	0.43**	0.24	0.49**
Communication	0.25	0.31	0.03	0.02	0.12	0.27	0.14	0.14
Mental Health	0.18	0.27	0.21	0.36*	0.39*	0.31	0.21	0.49**
Social functioning	0.26	0.47**	0.28	0.33	0.36*	0.60**	0.20	0.38*
Symptom frequency battery	0.19	0.38*	0.28	0.25	0.14	0.27	0.27	0.10

Spearman’s rho values are presented. Significant Spearman’s rho correlations are shown in bold print

The Bonferroni-corrected thresholds for statistical significance was set at 0.05/88 = 0.00057(*) and 0.001/88 = 0.000011(**)

There were no significant correlations found between Nor-SWAL-QOL subscales eating duration, fear of eating and communication and any of the SF-36 domains. Eating desire and symptom frequency battery subscales had non-significant correlations with all but one of the SF-36 domains.

In the principal component analysis three components with an eigenvalue greater than one were extracted. The first component (eigenvalue = 4.58, explaining 45.77% of the total variance), The second and third components (eigenvalue of 1.28 and 1.05, explaining 12.8 and 10.5% of the total variance respectively). The original dysphagia-specific subscales loaded on components 1 & 3, while general quality of life subscales loaded on component 2. These results differ from the 2 component loadings for dysphagia-specific and general quality of life items in the original SWAL-QOL. The results are shown in the Additional file 1.

#### Known-groups validity

Further assessment of construct validity includes determining if the Nor-SWAL-QOL can distinguish between persons with and without OD and be sensitive to clinically known features of OD. First, correlations of OD group and control group Nor-SWAL-QOL subscale scores were computed for known-groups validity using Mann-Whitney U. The effect size was determined to demonstrate clinical relevance for group comparisons. An effect size of *r* = 0.1 was considered small, *r* = 0.3 medium and *r* = 0.5 large [35]. The hypothesis that participants with OD would score lower (worse) than the control group participants on all subscales of the Nor-SWAL-QOL was confirmed as shown in Table 4. The mean scores for OD participants were significantly lower *(p < 0.001)* on all subscales, ranging from 40.0 to 65.6, while control group mean scores ranged from 86.5 to 100. Effect size, showing clinical relevance for group comparisons, was large for all subscales (0.62–0.91) except for the sleep subscale (0.45) [35]. Table 5 reveals that differences were also statistically significant *(p < 0.001)* between OD and control groups for all items on the symptom frequency battery, exhibiting sensitivity to clinically known features of OD. Effect size was again large between groups for all items on the symptom frequency battery.

**Table 4:** Construct validity; differences on the Nor-SWAL-QOL between oropharyngeal dysphagia (OD) and control group (known-groups validity)

Nor-SWAL-QOL	OD group		Control group		Mann-Whitney *U*	Effect size *r*
	*n*	Mean (SD)	*n*	Mean (SD)	Sign. (two-tailed)	
Burden	101	44.6 (28.2)	123	99.9 (1.1)	*p < 0.001*	0.91
Food selection	102	57.3 (31.7)	123	99.4 (3.9)	*p < 0.001*	0.81
Eating duration	102	40.0 (29.7)	122	98.9 (4.4)	*p < 0.001*	0.87
Eating desire	102	65.6 (28.9)	122	97.5 (8.8)	*p < 0.001*	0.73
Fear of eating	102	62.3 (27.6)	123	99.5 (2.3)	*p < 0.001*	0.83
Sleep	102	60.0 (31.0)	123	86.5 (18.5)	*p < 0.001*	0.45
Fatigue	101	52.9 (28.2)	123	87.5 (16.5)	*p < 0.001*	0.62
Communication	102	61.9 (33.0)	123	99.7 (2.5)	*p < 0.001*	0.76
Mental Health	102	46.3 (28.2)	123	99.9 (.902)	*p < 0.001*	0.91
Social functioning	102	54.4 (31.4)	123	100 (.000)	*p < 0.001*	0.85
Symptom frequency battery	99	55.6 (19.4)	123	97.5 (4.2)	*p < 0.001*	0.86

**Table 5:** Construct validity; differences in the Nor-SWAL-QOL between oropharyngeal dysphagia and control group scores on Symptom frequency battery

Nor-SWAL-QOL	OD group *(n = 102)*	Control group *(n = 123*	Mann Whitney *U* Sign. (two-tailed)	Effect size *r*
Symptom frequency battery	Mean (SD)	Mean (SD)		
Coughing	2.24 (1.0)	4.75 (0.5)	*p < 0.001*	0.85
Choke on food	3.50 (1.2)	4.96 (0.2)	*p < 0.001*	0.72
Choke on liquids	3.61 (1.2)	4.98 (0.1)	*p < 0.001*	0.69
Thick saliva, phlegm	2.56 (1.3)	4.76 (0.7)	*p < 0.001*	0.73
Gagging	3.72 (1.1)	4.94 (0.3)	*p < 0.001*	0.68
Drooling	3.41 (1.4)	4.92 (0.3)	*p < 0.001*	0.66
Problems chewing	3.68 (1.5)	4.97 (0.2)	*p < 0.001*	0.60
Excess saliva, phlegm	2.81 (1.4)	4.90 (0.4)	*p < 0.001*	0.75
Clear throat	2.47 (1.4)	4.66 (0.6)	*p < 0.001*	0.79
Food stick in throat	2.94 (1.1)	4.89 (0.4)	*p < 0.001*	0.80
Food stick in mouth	3.39 (1.3)	4.99 (0.1)	*p < 0.001*	0.75
Dribble from mouth	3.85 (1.3)	5.00 (0.0)	*p < 0.001*	0.62
Dribble from nose	4.29 (1.0)	5.00 (0.0)	*p < 0.001*	0.52
Cough food stuck	2.84 (1.3)	4.93 (0.3)	*p < 0.001*	0.81

### Sensitivity to dysphagia severity

Instrument items that address management strategies, such as the need for modified food and liquid consistency, use of a feeding tube for nutrition and the presence of symptoms on the symptom frequency battery, are considered indicators of dysphagia severity. Additional assessment of validity, to determine if the Nor-SWAL-QOL was sensitive to severity of the disorder was completed by computing the Kruskal-Wallis test for Nor-SWAL-QOL scores within the OD group, stratified according to types of food and liquid consistencies ingested, and for those tube fed or not tube fed. OD participants who ate pureed/blended consistencies had worse scores than those who ate regular consistencies on all Nor-SWAL-QOL subscales. These values were statistically significant for the subscales food selection, eating duration, eating desire, communication, social functioning and symptom frequency battery. Burden was near the 0.05 significance level cut off with *p = 0.052*. Adjusted statistical analyses revealed that the differences were between regular and pureed/blended for 5 of the subscales as shown in Table 6. Symptom frequency battery showed adjusted statistically significant values between regular and soft consistencies.

**Table 6:** Nor-SWAL-QOL sensitivity to severity by food consistency eaten^a^ and tube feeding status^b^

Nor-SWAL-QOL	Regular^a^ (*n* = 38)	Soft^a^ (*n* = 38)	Pureed/blended^a^ (*n* = 11)	Kruskal-Wallis Test Sign. *p* value	Kruskal-Wallis Test – pairwise comparisons Adj. sign. *p value*	Tube fed^b^ (*n* = 20)	Not tube fed^b^ (*n* = 81)	*p* value
	Mean (SD)	Mean (SD)	Mean (SD)			Mean (SD)	Mean (SD)	
Burden	53.9 (28.2)	44.6 (26.4)	32.1 (24.7)	*p=0.052*		25.2 (26.6)	49.6 (26.9)	p=0.001**
Food selection	71.0 (27.1)	56.1 (29.4)	33.1 (25.8)	p=0.001*	p=0.001**	41.4 (33.2)	61.6 (30.2)	p=0.016*
Eating duration	49.7 (29.7)	40.1 (28.0)	22.9 (23.8)	p=0.023*	p=0.022**	24.6 (29.2)	44.0 (28.9)	p=0.004*
Eating desire	79.0 (19.7)	66.3 (25.3)	56.0 (28.1)	p=0.011*	p=0.030**	40.0 (34.7)	71.6 (23.7)	p<0.001**
Fear	70.5 (22.2)	63.1 (26.2)	50.2 (31.1)	*p=0.145*		55.2 (34.6)	64.3 (25.6)	*p=0.326*
Sleep	62.0 (29.0)	60.4 (32.6)	58.2 (34.7)	*p=0.985*		52.8 (27.7)	61.4 (31.9)	*p=0.201*
Fatigue	57.7 (25.3)	50.0 (31.9)	50.8 (24.1)	*p=0.548*		45.0 (28.4)	54.8 (28.2)	*p=0.210*
Communication	73.1 (29.7)	60.4 (30.8)	44.4 (34.1)	p=0.023*	p=0.038**	44.6 (36.6)	66.5 (30.9)	p=0.017*
Mental health	53.1 (26.6)	46.3 (28.1)	31.4 (19.1)	*p=0.096*		35.0 (28.7)	49.0 (27.7)	*p=0.051*
Social functioning	64.9 (28.2)	55.4 (30.8)	37.0 (30.3)	p=0.033*	p=0.031**	30.0 (25.5)	60.3 (30.1)	p<0.001**
Symptom frequency battery	63.4 (13.2)	52.1 (19.3)	48.1 (21.9)	p=0.012*	p=0.030***	45.4 (22.4)	58.2 (17.9)	p=0.023*

^a^Food consistency sample limited to persons taking oral nutrition

*Statistically significant differences at *p*<0.05

** Adjusted statistically significant difference between regular and puree/blended

*** Adjusted statistically significant difference between regular and soft

^b^Tube feeding status

*Statistically significant differences at *p*<0.05 for food selection, eating duration, communication and symptom frequency battery

**Statistically significant differences at *p*<0.01 for burden, eating desire and social functioning

Significantly lower scores, corresponding to more severe problems, were also apparent on all subscales, except the symptom frequency battery, for ingestion of liquids. A statistically significant difference was only found on the subscale of eating desire; however it showed differences between both those who ingested thin liquids vs no liquids by mouth *(p = 0.013)*, and those who ingested thickened liquids vs no liquids by mouth *(p = 0.018).*

The Nor-SWAL-QOL sensitivity to severity was also evident when summarizing differences in subscales scores for OD participants that are tube fed or not tube fed, also shown in Table 6. Statistically significant values at *p < 0.05* were evident for food selection, eating duration, communication and symptom frequency battery, while subscales for burden, eating desire and social functioning were statistically significant at *p < 0.01*. Mental health significance of *p = 0.051* was near cut off for statistical significance at *p < 0.05*.

## Discussion

The use of valid and reliable measurements is essential for providing evidence-based health care. Treatment in OD is often based solely on measurements of physiological function, however as McHorney noted, “…physiologic function is not synonymous with patient functioning and well-being” [19]. A patient reported outcome measure (PROM) is “any report of the status of a patient’s health condition that comes directly from the patient, without interpretation of the patient’s response by a physician or anyone else” [40]. The use of more disease-specific health-related quality of life (HRQoL) instruments, intended to reflect the patients self-perceived health status in relation to a specific condition, has become increasingly common practice to reveal patients experiences with OD and treatment effect in OD management [41]. Several systematic reviews refer to the Swallowing Quality of Life (SWAL-QOL) questionnaire as superior and the gold standard for use with diverse populations suffering from dysphagia [42–45].

There is currently no standardized or validated dysphagia-specific QoL measurement available in Norway for use in evidence-based assessment and treatment. This study evaluated the psychometric properties of the translated and culturally adapted Nor-SWAL-QOL.

There was a high response rate and positive response from persons having OD to this study. The study included persons from a wide spectrum of etiologies, the majority with neurological etiologies which is known as a major cause of OD in adults. A uniqueness of this study is the wide range of years that participants had lived with OD and the severity level of OD. Ninety percent of the participants had lived with OD for more than 1 year; with nearly 20% of these living with OD for more than 11 years, and a majority (62%) reported the need for modified consistency or were tube fed. The original SWAL-QOL and other validation studies had not reported length of time participants had OD or had a majority of participants with mild severity [18, 22–28]. This information supports the Nor-SWAL-QOL validation as having a good representation of the population.

Although age and gender differences were significant between the OD group and control group, weighting adjustment revealed that these differences would not have an effect on analysis results. The majority (77%) of the OD group had a high school education or above, however they had fewer years of education compared to the control group. Nevertheless, the Norwegian translation was kept as similar to the original as possible, which was developed using language appropriate for elementary school reading level. There were neither reports from pilot-testing nor comments from study participants on difficulty with understanding the questions.

Full score distribution range (0–100) was met for all 10 subscales, only eating duration had floor effects exceeding the recommended 15% (16.7%) which was similar to original SWAL-QOL results and likely emphasize how lengthy mealtimes are a common problem in this population. Food selection, eating desire, sleep and communication subscales had ceiling effects which were also similar to, but generally lower than the original SWAL-QOL results. A possible explanation for ceiling effects for these subscales may be that most participants in this study group had been living with their OD for many years, thus more likely to know what foods and liquids they can manage, had discovered new foods which help retain the desire to eat and experienced improved communication skills. According to McHorney, these floor and ceiling effects should not be of great concern as it should still be possible for patients to reveal both positive and negative change in other subscales following treatment [18].

Evidence of construct validity, agreement between different measures of similar constructs, in this case QoL, was seen with significant correlations between Nor-SWAL-QOL subscales; fatigue, sleep, social functioning and mental health and the SF-36 subscales; general health, vitality, social functioning and mental health. Fatigue and sleep subscales are general QoL subscales, therefore expected to have similarities with the SF-36 domains general health and vitality. Similar expectations were supported by the higher correlations between social functioning and mental health subscale of both instruments. These results support convergent validity of the Nor-SWAL-QOL. Conversely, discriminant validity was demonstrated by non-significant and weak correlations seen between the Nor-SWAL-QOL subscales; eating duration, eating desire, fear of eating, communication and the symptom frequency battery with nearly all of the SF-36 domains. The low level of correlation between these dysphagia-specific constructs of the Nor-SWAL-QOL and SF-36 domains, which measures general QoL, provides good evidence that these two instruments measure different constructs which gives strong support to the validity of Nor-SWAL-QOL.

PCA revealed that the Nor-SWAL-QOL items loaded on three principle components for this study sample instead of 2 components as with the original SWAL-QOL. Food selection and eating desire loaded strongly on the third component. Contrary, mental health and social functioning did not load strongly onto one component. However, those components together (food selection, eating desire, mental health and social functioning) could be considered to represent more of the psychosocial variance of the Nor-SWAL-QOL than the original SWAL-QOL.

Mean score differences on the Nor-SWAL-QOL for OD and control groups were statistically significant on all 10 subscales and all symptom frequency battery items exemplifying known-groups validity.

Additionally, Nor-SWAL-QOL demonstrated statistically significant differences within the OD group, both for those requiring food and liquid modifications and for those who are tube fed or not tube fed. There were significant differences for five and six of the ten dysphagia-specific subscales and the symptom frequency battery between OD participants who ate regular food and those who ate pureed food, and those who were tube-fed or not tube-fed respectively, thus supporting the hypothesis that dysphagia severity, as demonstrated by the need for adjustments in food consistency or feeding tube dependency, is associated with reduced QoL. It was noteworthy that although there were 20 participants that reported receiving food/liquid via a feeding tube, not one was recorded as scoring a zero on the FOIS. This may indicate that the practice of ‘nothing by mouth’ is non-existent in this sample.

Reliability estimates of internal consistency for all subscales and the symptom frequency battery were adequate for the Nor-SWAL-QOL (Cronbach’s α ≥0.70). The symptom frequency battery and all subscales, with the exception of eating duration and eating desire reached the recommended Cronbach’s α cut off of ≥0.80 for group level research. Lower estimates for these two subscales were similar to other culturally adapted versions of the SWAL-QOL [22, 23, 28]. A possible explanation for this may be the original sentence formulation used for these subscales including double negatives and discrepancies with translation. Test-retest reliability estimates for short term stability and Intraclass correlations (ICC) for the Nor-SWAL-QOL were moderate to good [18].

There is an emergence in the use of the item response theory (IRT) approach to improve the development and analysis of the psychometric properties of PROM’s for OD [46, 47]. However, the purpose of this study was to validate the original SWAL-QOL in a Norwegian population. The IRT approach will be implemented in future development of PROM’s for this population.

### Limitations

A limitation of the SWAL-QOL mentioned in previous validation studies, is the large number of questions and amount of time needed to complete the survey. The majority of OD participants in this study reported using between 15 and 30 min to complete the Nor-SWAL-QOL, which is longer than the original SWAL-QOL survey averaging 14 min and for other the validations mentioned previously. In addition, many participants in this study required assistance in filling out the questionnaire (41%). These findings, increased time filling out the questionnaire and need for help, may be a reflection of the severity level of OD group as mentioned above.

The appropriateness for use with tube-fed patients was mentioned as a possible limitation in the original SWAL-QOL and other translations [24, 28, 29]. In this study, 20% of the participants were tube-fed to some extent and although there were a few comments on the applicability of some questions, tube-fed participants did not have a greater number of missing items than non-tube fed. This may be because the majority of tube-fed patients in this study had neurological diagnosis and received help completing the questionnaire as compared to others, such as head and neck participants.

## Conclusion

Use of both objective physiologic outcome measures and disease-specific HRQoL provide two unique and valuable perspectives resulting in the best overall evaluation of the impact OD on a person’s life. The devastating medical complications and psychosocial effects of OD on quality of life necessitate access to valid and reliable dysphagia-specific QoL outcome measures for health care practitioners, dysphagia clinicians and researchers. There is currently no such validated instrument in Norway. This cultural validation study revealed that the Norwegian version of SWAL-QOL demonstrated acceptable convergent and discriminant validity, distinguished between persons with and those without oropharyngeal dysphagia, showed sensitivity to clinically known features of oropharyngeal dysphagia, was able to differentiate between symptom severity levels, and exhibited adequate reliability in this study sample. In summary, the Nor-SWAL-QOL demonstrated good to excellent psychometric properties and will be a valuable tool in the assessment and treatment of individuals living with oropharyngeal dysphagia.

## Supplementary information

**Additional file 1: Table S1.** Descriptive characteristics for oropharyngeal dysphagia (OD) and control groups. **Table S2.** Score distribution on Functional Oral Intake Scale. **Figure S1.** Principal Component Analysis (PCA) of Nor-SWAL-QOL; KMO, Bartlett’s, Scree Plot, orthogonal and oblique rotation.

## Data Availability

The datasets analyzed in the current study are available from the corresponding author upon reasonable request.

## Abbreviations

FOIS: Functional Oral Intake Scale
HRQoL: Health Related Quality of Life
ICC: Intraclass correlation coefficient
MASA: Mann Assessment of Swallowing Ability
Nor-SWAL-QOL: Norwegian version of SWAL-QOL
OD: Oropharyngeal dysphagia
PROM: Patient-reported outcome measure
QoL: Quality of life
SF-36: Short Form 36
SWAL-QOL: Swallowing Quality of Life questionnaire
US-FDA: U.S. Food and Drug Administration
VFSS: Videofluoroscopic swallow study
