# Supplementary material for: Cultural adaptation and validation of the Norwegian version of the swallowing quality of life questionnaire (SWAL-QOL)
Source: Health Qual Life Outcomes. 2019 Dec 5;17:179. doi: 10.1186/s12955-019-1248-0 (PMC6896756; doi:10.1186/s12955-019-1248-0)
Supplement: Supplementary file 1 — Additional file 1: Table S1. Descriptive characteristics for oropharyngeal dysphagia (OD) and control groups. Table S2. Score distribution on Functional Oral Intake Scale. Figure S1. Principal Component Analysis (PCA) of Nor-SWAL-QOL; KMO, Bartlett’s, Scree Plot, orthogonal and oblique rotation. [file 12955_2019_1248_MOESM1_ESM.docx]

**Table S1.** Descriptive characteristics for oropharyngeal dysphagia (OD) and control groups

| **Characteristic** | **OD group** | **%** | **Control group** | **%** | **T-test**  **Sig. two- tailed** |
| --- | --- | --- | --- | --- | --- |
| **Gender** | *(n=102)* |  | *(n=123)* |  |  |
| Male | 59 | 57.8 | 53 | 43.1 | *p=0.028* |
| Female | 43 | 42.2 | 70 | 56.9 |  |
| **Age (years)** | *(n=102)* |  | *(n=123)* |  |  |
| Range | 24-87 |  | 18-92 |  |  |
| Mean (SD) | 60.0 (14.0) |  | 54.6 (15.4) |  | *p=0.002* |
| Median | 62 |  | 56 |  |  |
| **Reason for dysphagia** | *(n=102)* |  |  |  |  |
| Cerebral vascular accident | 28 | 27.5 |  |  |  |
| Traumatic brain injury | 7 | 6.9 |  |  |  |
| Neurodegenerative disorder | 9 | 8.8 |  |  |  |
| Other neurological disorder | 15 | 14.7 |  |  |  |
| Head and Neck cancer | 15 | 14.7 |  |  |  |
| Unknown | 10 | 9.8 |  |  |  |
| Other | 18 | 17.6 |  |  |  |
| **Education** | *(n=99)* |  | *(n=122)* |  | *p* <0.001 |
| Elementary school | 5 | 5.0 |  |  |  |
| Middle school | 20 | 20.2 | 1 | .8 |  |
| High school | 39 | 39.4 | 20 | 16.4 |  |
| University and above | 38 | 38.4 | 101 | 82.8 |  |
| **Assistance filling out questionnaire** | *(n=100)* |  | *(n=123)* |  |  |
| Read or write | 36 | 36 |  |  |  |
| Answered for me | 2 | 2.0 |  |  |  |
| Other type of help | 3 | 3.0 |  |  |  |
| No assistance | 59 | 59.0 | 123 | 100 |  |
| **Time used to fill out questionnaire** | *(n=99)* |  | *(n=122)* |  |  |
| < 15 minutes | 23 | 23.2 | 85 | 69.7 |  |
| 15-30 minutes | 50 | 50.5 | 32 | 26.2 |  |
| 30-45 minutes | 17 | 17.2 | 4 | 3.3 |  |
| 45-60 minutes | 7 | 7.0 | 1 | .8 |  |
| >60 minutes | 2 | 2.0 |  |  |  |
| **Tube fed** | *(n=101)* |  |  |  |  |
| Yes | 20 | 19.8 |  |  |  |
| No | 81 | 80.2 |  |  |  |
| **Consistency of food eaten mostly** | *(n=101)* |  | *(n=123)* |  |  |
| Normal | 38 | 37.6 | 123 | 100 |  |
| Soft/modified | 38 | 37.6 |  |  |  |
| Puree/blender | 11 | 10.9 |  |  |  |
| Most in tube | 6 | 5.9 |  |  |  |
| Everything in tube | 8 | 7.9 |  |  |  |
| **Consistency of liquids** | *(n=101)* |  | *(n=123)* |  |  |
| Thin | 80 | 79.2 | 123 | 100 |  |
| Slightly thick | 9 | 8.9 |  |  |  |
| Moderately thick | 5 | 5.0 |  |  |  |
| Very thick/pudding | 2 | 2.0 |  |  |  |
| No liquids by mouth | 5 | 5.0 |  |  |  |
| **General health** | *(n=100)* |  | *(n=121)* |  |  |
| Poor | 25 | 25.0 | 0 |  |  |
| Fair | 35 | 35.0 | 5 | 4.1 |  |
| Good | 27 | 27.0 | 23 | 18.7 |  |
| Very good | 11 | 11.0 | 52 | 42.3 |  |
| Excellent | 2 | 2.0 | 41 | 33.3 |  |
| **Time period from debut of oropharyngeal dysphagia and SWAL-QOL in years** | *(n=102)* |  |  |  |  |
| < 12 months | 10 | 9.8 |  |  |  |
| 2-5 years | 51 | 50 |  |  |  |
| 6-10 years | 21 | 20.6 |  |  |  |
| > 11 years | 20 | 19.6 |  |  |  |

**Table S2.** Score distribution on Functional Oral Intake Scale

| **Functional Oral Intake Scale level** | **Level description** | ***OD group***  ***(n=102)*** | **Score distribution (%)** |
| --- | --- | --- | --- |
|  | **Tube dependent (levels 1-3)** | | |
| 1 | Nothing by mouth | 0 | 0 |
| 2 | Tube-dependent with minimal/inconsistent oral intake | 8 | 7.8 |
| 3 | Tube supplements with consistent oral intake | 9 | 8.8 |
|  | **Total oral intake (levels 4-7)** | | |
| 4 | Total oral intake of a single consistency | 1 | 1.0 |
| 5 | Total oral intake of multiple consistencies requiring special preparation | 16 | 15.7 |
| 6 | Total oral intake with no special preparation, but must avoid specific foods or liquid items | 41 | 40.2 |
| 7 | Total oral intake with no restrictions | 27 | 26.5 |

**Figure S1.** Principal Component Analysis (PCA) of Nor-SWAL-QOL; KMO, Bartlett’s, Scree Plot, orthogonal and oblique rotation


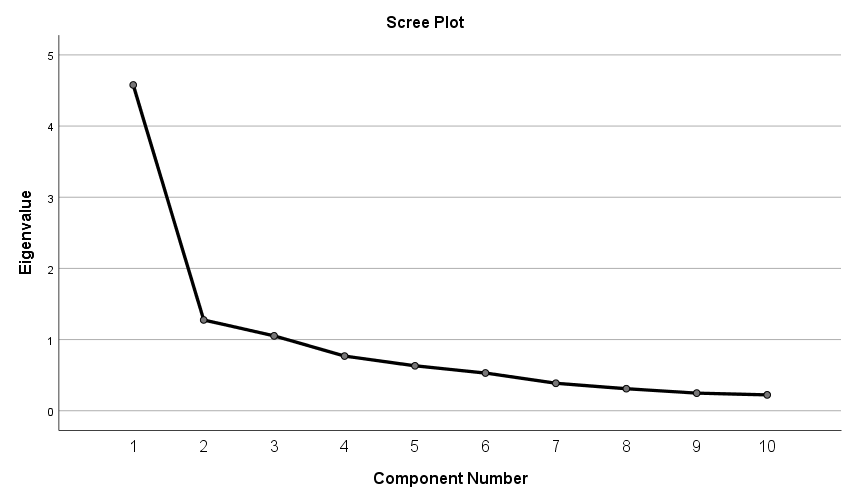


| Kaiser-Meyer-Olkin Measure of Samplin Adequacy | | 0.805 |
| --- | --- | --- |
| Bartlett’s Test of  Sphericity | Approx.. Chi-Square | 428.542 |
|  | df | 45 |
|  | Sig. | .000 |

**KMO and Bartlett’s Test**

| Pattern Matrix^a^ | | | |
| --- | --- | --- | --- |
|  | Component | | |
|  | 1 | 2 | 3 |
| Communication | **0.88** |  | 0.15 |
| Fear of eating | **0.68** | -0.22 |  |
| Eating duration | **0.66** | 0.30 | -0.31 |
| General burden | **0.52** | -0.29 | -0.26 |
| Sleep |  | **-0.91** |  |
| Fatigue |  | **-0.80** | -0.19 |
| Social functioning | 0.29 | -0.45 | -0.32 |
| Eating desire |  |  | **-0.91** |
| Food selection |  | -0.17 | **-0.86** |
| Mental health | 0.38 | -0.28 | -0.41 |
| Extraction Method: Principal Component Analysis.  Rotation Method: Oblimin with Kaiser Normalization^.a^  a. Rotation converged in 9 iterations. | | | |

| Rotated Component Matrix^a^ | | | |
| --- | --- | --- | --- |
|  | Component | | |
|  | 1 | 2 | 3 |
| Communication | **0.82** | 0.11 |  |
| Eating duration | **0.67** | -0.14 | 0.40 |
| Fear of eating | **0.66** | 0.32 |  |
| General burden | **0.58** | 0.42 | 0.38 |
| Sleep |  | **0.88** |  |
| Fatigue | 0.17 | **0.83** | 0.27 |
| Social functioning | 0.39 | 0.54 | 0.41 |
| Eating desire | 0.17 |  | **0.87** |
| Food selection | 0.10 | 0.29 | **0.83** |
| Mental health | 0.48 | 0.40 | 0.50 |
| Extraction Method:Principal Component Analysis  Rotation Method: Varimax with Kaiser Normalization^a^  a. Rotation converged in 5 iterations | | | |
